# Is there an advantage to delivering breast boost in the lateral decubitus position?

**DOI:** 10.1186/1748-717X-7-163

**Published:** 2012-09-24

**Authors:** Neeta Kannan, Peyman Kabolizadeh, Hayeon Kim, Christopher Houser, Sushil Beriwal

**Affiliations:** 1 Department of Radiation Oncology, University of Pittsburgh Cancer Institute, Pittsburgh, PA, 15213, USA

**Keywords:** Breast cancer, Body mass index, Electron beam therapy

## Abstract

**Background:**

The purpose of this study was to compare the change in depth of target volume and dosimetric parameters between the supine and lateral decubitus positions for breast boost treatment with electron beam therapy.

**Methods:**

We analyzed 45 patients who were treated, between 2009–2010, with whole breast radiation (WBRT) followed by a tumor bed boost in the lateral decubitius position. Tumor bed volume, distance from skin to the maximal depth of the tumor bed, D90 (dose covering 90% of the tumor bed volume), maximal dose, electron energy and doses to heart and lungs were compared. Additional variables of body mass index (BMI) and tumor bed location were also analyzed to see if there was a benefit limited to any subgroup.

**Results:**

Median BMI for the 45 patients treated was 30.6 (20.6-42.4). When comparing the supine scan to the lateral decubitus scan, there was no significant difference in the tumor bed volume (p = 0.116). There was a significant difference between depth to the tumor bed in the supine scan and lateral decubitus scan (p < 0.001). The mean maximum doses and D90 between the two scans were 110.7 (100.0-133.0)% vs 106.1 (95.1-116.9)% (p < 0.05) and 93.9 (81.3-01.0-101.0)% vs. 98.2 (89.1-108.0)% (p = 0.004) respectively. There was no difference in dose delivered to the lungs or heart between the two scans (p = 0.848 and p = 0.992 respectively). On subset analysis, there was a difference in depth to tumor that was seen across all BMI classes, including normal (p ≤ 0.001, overweight (p ≤ 0.001) and obese (p ≤ 0.001). The majority of patients had a tumor in the upper outer quadrant (77.8%) and on subset analysis, there was a significant difference in tumor bed volume (p < 0.01), depth to tumor (p < 0.01), tumor bed coverage [D90] (p < 0.05), maximum dose (p < 0.05) and energy (p < 0.001) for this location.

**Conclusions:**

Delivering a tumor bed boost in the lateral decubitus position reduces the distance to the tumor bed allowing for a lower energy treatment to be used to treat breast cancer. It improves coverage and decreases maximal dose to the target volume, all of which would help reduce skin morbidities and should be considered for patients with upper outer quadrant disease, irrespective of BMI status.

## Background

Breast-Conserving Therapy (BCT) progressed to be widely accepted since the 1980s, when several randomized trials showed similar survival rates for the patients who received radical mastectomy and those who received BCT. Breast-Conserving Therapy followed by Whole Breast Radiation Therapy (WBRT) to treat stage I or II breast cancer, has been shown to reduce the risk of local recurrence [1-3]. The National Surgical Adjuvant Breast and Bowel Project (NSABP) trial B-06 has shown a reduction in ipsilateral breast recurrence from 39.2% to 14.3% when treating patients with BCT followed by WBRT vs. BCT alone [1]. Since then, studies have shown that an additional boost to the tumor bed after WBRT can decrease the local recurrence rates and failures [4-6]. The European Organisation for Research and Treatment of Cancer (EORTC) 22881–10882 has shown that relative risk reduction of local recurrence by the boost in addition to WBRT was significant in all age groups, while the absolute risk reduction at 10 years was larger in younger patients (patients <40 years old). However, addition of boost was associated with poor cosmetic results. The cosmetic results were scored as excellent to good in 86% of the patients receiving no boost and in 71% of the patients receiving a boost. Severe fibrosis at 10 years was 4.4% in patients receiving boost verses 1.6% in patients receiving no boost. Apart from the boost, other predictors of poor cosmetic outcome were identified. In the nomogram developed by Collette et al. [7] for predicting breast fibrosis following a boost dose, the treatment factors such as higher dose, photon or interstitial implantation, and energy of electrons were found to be of significance.

There are several techniques that can be used to deliver the breast boost including electron beam, photon beam, or interstitial implantation with the electron beam being the most common technique used. However, there are limitations involved in using electron beam technique, given that the dose delivery to the distal surgical bed may be limited by the depth of surgical bed or patient’s body habitus. Different techniques have been used including prone decubitus positioning and recently the lateral decubitus positioning to treat the boost volume [8]. In lateral decubitus positioning technique, placing the patient in the lateral decubitus position with the patient rotated over the uninvolved breast can result in a reduction in the distance from the skin to the target volume maximum depth, particularly in patients with a body mass index (BMI) ≥ 30 [9]. This may result in using lower electron energy and ultimately decreasing the fibrosis. In this study, the depth of target volume in the lateral decubitus position was analyzed in association with the corresponding changes in the dosimetric parameters and was compared with supine position to evaluate the potential advantage of lateral decubitus position.

## Methods

A retrospective study was conducted on 100 patients, who had undergone WBRT followed by tumor bed boost. Of these, 45 patients were treated in the lateral decubitus position between 2009–2010 and were subjects of the study. All these patients were originally treated with CT-based photon plan for whole breast external beam radiation therapy (WBRT) in the supine position and underwent re-simulation in the lateral decubitus position for boost treatment. A comparison of the plan for electron boost was made between original supine and lateral decubitus position scans. A lateral decubitus scan was obtained 4 or 5 weeks after starting WBRT. To make the lateral decubitus scan reproducible, a customized vacuum bag was made. The dosimetric parameters including surgical bed volume, distance from skin to maximal depth of the surgical bed, D90 (the dose covering 90% of the tumor bed volume), maximal dose, electron energy used, and doses to the heart (if the left breast was irradiated) and ipsilateral lung were recorded in the supine and lateral decubitus scans. Additional variables of BMI and tumor bed location were also analyzed to evaluate whether there was a benefit particular to any subgroup.

## Results

The median BMI for the 45 patients treated was 30.6 (20.6-42.4). Of this sample population, 24 had tumor in the left breast, and the remaining 21 had tumor in the right breast. Furthermore, 35 patients had tumor located in the upper outer quadrant, seven had tumor in the lower outer quadrant, two had tumor in the upper inner quadrant, and one patient had tumor in the lower inner quadrant. Median time between the supine and lateral decubitus scans was 37 (28–62) days.

Results are summarized in Tables 1, 2, 3, 4. The mean surgical bed volumes for the supine and lateral decubitus scans were 31.2 cc (2.5-198) and 25.8 cc (3.01-277.39) respectively, without a significant difference in the volumes (p = 0.116) between the two scans. However, there was a significant difference between maximum depth of tumor bed in the supine scan and lateral decubitus scan (p < 0.001), with mean values of 5.7 cm (3.0-9.0) and 3.56 cm (1.1-6.4) respectively. The mean energy used in the supine and lateral decubitus scan was 18 MeV (12–20) and 13.6 MeV (9–20), (p < 0.001) respectively. The mean maximum doses and D90 between the two scans were 110.7% versus 106.1% (p < 0.05) and 93.9% versus 98.2% (p = 0.004) respectively. There was no difference in dose delivered to the lungs or heart between the two scans (p = 0.848 and p = 0.992 respectively) [Table 1].

**Table 1:** Dosimetric parameters for all patients

**Mean (St. deviation)**	**Original**	**Lateral decubitus**	**P-value**
**Tumor Bed (cc)**	31.2 (2.5-198.0)	25.8 (3.0-277.4)	0.116
**Depth to Tumor (cm)**	5.7 (3.0-9.0)	3.56 (1.1-6.4)	**0.000**
**D90 (%)**	93.9 (81.3-101.0)	98.2 (89.1-108.0)	**0.004**
**Maximum Dose (%)**	110.7 (100.0-133.0)	106.1 (95.1-116.9)	**0.04**
**Lung V50 (%)**	2.2 (0.0-14.4)	2.3 (0.0-19.6)	0.848
**Heart Max (Gy) (N = 24)**	4.6 (0.4-13.7)	4.6 (0.1-11.2)	0.992
**Energy (Mev)**	18 (12–20)	13.6 (9–20)	**0.000**

**Table 2:** Dosimetric parameters of patients with tumor volume difference <3.0 cc between original and lateral decubitus scans

**Mean (St. deviation), N = 17**	**Original**	**Lateral decubitus**	**P-value**
**Tumor Bed (cc)**	9.5 (5.0)	9 (5.4)	0.172
**Maximum Depth of Tumor Bed (cm)**	5.2 (1.2)	3.2 (0.7)	**0.000**
**D90 (%)**	94.1 (5.0)	98.3 (6.0)	0.091
**Maximum Dose (%)**	110.5 (9.6)	105.5 (5.9)	0.118
**Lung V50 (%)**	0.52 (1.1)	0.71 (1.1)	0.479
**Heart Max (Gy) (N = 10)**	3.7 (4.0)	4.6 (4.3)	0.451
**Energy (Mev)**	16.5 (3.1)	12.7 (3.2)	**0.000**

**Table 3:** Dosimetric parameters based on BMI class

**Parameter (Mean)**	**Position**	**BMI < 25**	**p-value**	**BMI 25-30**	**p-value**	**BMI >30**	**p-value**
**Tumor Bed (cc)**	Original	15.7	0.155	36.9	0.935	33.4	0.094
	Lat. Decub.	15.8		36.3		25.3	
**Depth to rumor (cm)**	Original	4.8	0.000	5.2	0.001	6.3	0.000
	Lat. Decub.	2.8		3.4		3.9	
**D90 (%)**	Original	95.5	0.922	95.1	0.592	92.7	0.001
	Lat. Decub.	95.7		97.2		108.1	
**Maximum Dose (%)**	Original	110.9	0.160	112.2	0.069	109.9	0.121
	Lat. Decub.	103.9		106.0		106.8	
**Lung V50 (%)**	Original	4.3	0.808	2.1	0.342	1.5	0.791
	Lat. Decub.	3.8		2.9		1.6	
**Heart Max (Gy)**	Original	6.9	0.437	5.2	0.584	2.8	0.304
	Lat. Decub.	5.46		4.4		4.4	
**Energy (Mev)**	Original	15.5	0.242	16	0.001	18.3	0.001
	Lat. Decub.	11.1		12.5		15.0	

**Table 4:** Dosimetric parameters of patients with upper outer quadrant tumor

**Mean (St. deviation), N = 35**	**Original**	**Lateral decubitus**	**P-value**
**Tumor Bed (cc)**	24.6 (24.2)	17.9 (21.8)	**0.007**
**Depth to Tumor (cm)**	5.7 (1.7)	3.4 (1.0)	**0.000**
**D90 (%)**	93.8 (4.7)	97.5 (5.5)	**0.024**
**Maximum Dose (%)**	110.7 (7.6)	106.0 (5.6)	**0.022**
**Lung V50 (%)**	1.8 (3.1)	2.1 (3.9)	0.517
**Heart Max (Gy) (N = 19)**	4.0 (3.6)	4.5 (3.8)	0.588
**Energy (Mev)**	16.8 (3.2)	13.0 (3.7)	**0.000**

To account for changes in surgical bed volume between the two scans, plan was separately evaluated among 17 patients, for whom the change in the surgical bed volume was less than 3.0 cc [Table 2]. There remained a significant difference in maximal depth of the tumor bed between the supine and lateral scans measuring 5.2 cm (3.7-7.6) versus 3.2 cm (1.8-4.3) respectively (p < 0.001). The mean energy used in the supine and lateral decubitus scan was also significantly Different (p < 0.001).

On subset analysis, there was a significant difference seen in maximum depth to tumor bed across all BMI categories, including normal (p < 0.001), overweight (p ≤ 0.001), and obese (p < 0.01) [Table 3]. Because the majority of patients had a tumor in the upper outer quadrant (77.8%), a subset analysis of this region was conducted and showed a significant difference in tumor bed volume (p < 0.01), maximum depth of tumor bed (p < 0.01), tumor bed coverage [D90] (p < 0.05), maximum dose (p < 0.05) and electron energy used (p < 0.001) between the two scans [Table 4].

## Discussion

**Figure 1:**
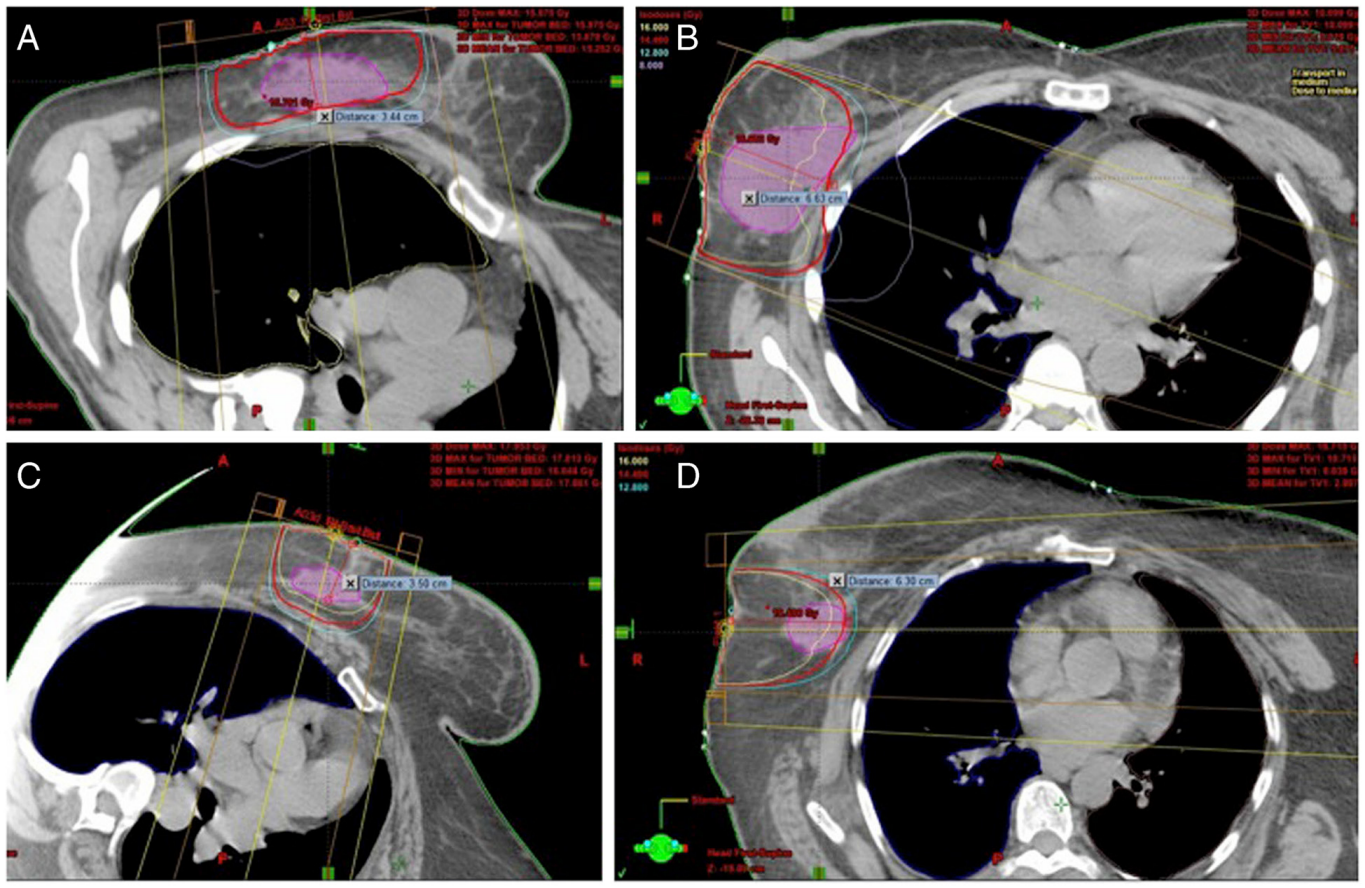
**Representative beam arrangement, beam energy and coverage in lateral decubitus versus supine positioning in two different patients.** (**A**) 12 MeV beam energy resulted in an adequate coverage in the lateral decubitus position in patient A. (**B**) 20 MeV beam energy was needed to reach an adequate coverage when patient A in supine position. 90% isodose line-14.4 Gy -was highlighted to review the coverage. (**C**) 16 MeV beam energy resulted in an adequate coverage in the lateral decubitus position in patient B. (**D**) 20 MeV beam energy did not result in an adequate coverage of the surgical bed when patient B in supine position. 90% isodose line-14.4 Gy -was highlighted to review the coverage.

Boost dose after whole breast radiation therapy improves local control at the cost of decreased cosmesis. The factors that predict poor cosmetic results using electron beam are dosage and higher energy [7]. In our series, re-simulation of selected patients in lateral decubitus position for breast boost treatment resulted in decreasing the maximum distance from the skin to the surgical bed, which facilitated the use of lower electron energies and decreased maximal dose to the target volume [Figure 1. These two gains have the potential to improve cosmetic results.

Moreover, some patients with supine scan had maximal depth of surgical bed to more than 6 cm, which was reduced significantly in lateral decubitus position. These patients were not suitable for electron beam boost and would have to be treated with conformal photon beams. Photon beam techniques increase the integral dose to breast in addition to increasing the volume of breast tissue treated to the prescribed dose. This has shown to result in higher likelihood of poor cosmetic results in EORTC trial.

Study by Ludwig et al. [9] on 231 patients who underwent re-simulation in the lateral decubitus position has shown that the lateral decubitus positioning reduces the distance from skin to maximal target volume depth in all patients. Average depth reduction by repositioning was 2.12 cm, allowing for an average electron energy reduction of approximately 7 MeV. Furthermore, mean skin entrance dose was reduced from about 90% to about 85% (p < 0.001). Also, if treated in the original supine position, 146 (63.2%) of the patients would not have the target volume covered by the distal 90% isodose line of the maximum electron energy available at their institution. Of the patients studied, 14 (6%) experienced moist desquamation in the boost field at the end of treatment. Average BMI of these patients was 30.4 (17.8–50.7). BMI greater than 30 was associated with more depth reduction by repositioning and increased risk of moist desquamation. These results were validated in our study. Moreover, the maximal dose to the target volume was also reduced with this technique, which results in a better homogeneity. In addition, repositioning provides a flat surface for optimum electron dosimetry, with an electron plane perpendicular to the surface.

In our study, patients were grouped based on body mass index (BMI), and the analysis showed a significant difference between supine and lateral decubitus scans in term of maximum depth of tumor bed for all BMI classes. In contrast, the study by Ludwig et al. [9] reported that BMI above 30 was associated with an increased risk of moist desquamation and a more depth reduction by lateral decubitus repositioning. The author suggested that the low moist desquamation rate in the boost field with lateral decubitus positioning implies that the boost treatment is well tolerated in patients with high BMI, who would have a higher risk of toxicity with conventional boost plan. This difference can be attributed to the fact that in Ludwig’s series this technique was used selectively (9.4% of all patients who had boost radiation therapy) and there may have been selection bias in using this technique to treat more patients with higher BMI. In contrast, at our institution this technique is used in about 45% of the patients who need boost. On subgroup analysis we found that lateral decubitus positioning would result in a shorter maximum depth to the tumor bed, better D90, lower D-max, and allowing the usage of lower electron energies in patients with upper outer quadrant tumors. However, specific comments cannot be made regarding any difference between patients with different quadrant tumors, given the small number of patients with disease in other quadrants. It is also important to highlight that in patients with significant seroma, the re-simulation can account for changes in its size over time and, therefore, result in a better boost plan, taking into account the new breast anatomy. Moreover, it is important to mention that electron energy is one of many factors that determines cosmetic outcome. These other factors may include surgical technique, total radiation dose and fraction size, radiation technique and any genetic predisposition to late side effects.

## Conclusions

In conclusion, using the lateral decubitus positioning for breast boost treatment in selected patients results in reducing the maximum depth to tumor bed, which facilitates the use of lower electron energy and increases the likelihood of using boost via electron beam technique. It also helps to reduce the maximal dose and improve the target volume coverage (Figure 1). These improvements can lessen the acute skin reactions and also help reduce the fibrosis, thus improving the cosmetic results. Patients with upper outer quadrant tumors may benefit more from lateral decubitus positioning than other patients. Therefore, it is important to choose patients for re-simulation when the benefit of this technique is maximized based on the above-mentioned factors. Future directions would include analyzing the clinical outcome in patients treated in lateral decubitus position, particularly in terms of late effects and cosmetic results.

## Abbreviations

BCT: Breast conserving therapy; BMI: Body mass index; D90: Dose covering 90% of the tumor bed volume; EORTC: The European Organisation for Research and Treatment of Cancer; NSABP: National Surgical Adjuvant Breast and Bowel Project; WBRT: Whole breast radiation therapy.

## Competing interests

The authors have no financial or personal relationships with other people or organizations that could inappropriately influence this work.

## Authors' contributions

NK was responsible for data collection & manuscript writing. PK helped with manuscript writing. HK and CH were responsible for planning and collection of data. SB was responsible for concept design, analysis, and writing manuscript. All authors read and approved the final manuscript.
